# Structural Insights of Three 2,4-Disubstituted Dihydropyrimidine-5-carbonitriles as Potential Dihydrofolate Reductase Inhibitors

**DOI:** 10.3390/molecules26113286

**Published:** 2021-05-29

**Authors:** Lamya H. Al-Wahaibi, Althaf Shaik, Mohammed A. Elmorsy, Mohammed S. M. Abdelbaky, Santiago Garcia-Granda, Subbiah Thamotharan, Vijay Thiruvenkatam, Ali A. El-Emam

**Affiliations:** 1 Department of Chemistry, College of Sciences, Princess Nourah bint Abdulrahman University, Riyadh 11671, Saudi Arabia; lhalwahaibi@pnu.edu.sa; 2 Discipline of Chemistry, Indian Institute of Technology, Gandhinagar 382355, India; althaf.shaik@iitgn.ac.in; 3 Department of Pharmaceutical Organic Chemistry, Faculty of Pharmacy, Mansoura University, Mansoura 35516, Egypt; mwahhab95@gmail.com; 4 Department of Physical and Analytical Chemistry, Faculty of Chemistry, Oviedo University-CINN, 33006 Oviedo, Spain; saidmohammed.uo@uniovi.es (M.S.M.A.); sgg@uniovi.es (S.G.-G.); 5 Biomolecular Crystallography Laboratory, Department of Bioinformatics, School of Chemical and Biotechnology, SASTRA Deemed University, Thanjavur 613401, India; thamu@scbt.sastra.edu; 6 Discipline of Biological Engineering, Indian Institute of Technology Gandhinagar, Gujarat 382355, India; vijay@iitgn.ac.in; 7 Department of Medicinal Chemistry, Faculty of Pharmacy, Mansoura University, Mansoura 35516, Egypt

**Keywords:** pyrimidine-5-carbonitriles, dihydrofolate reductase, crystal structure, DFT, Hirshfeld surface analysis

## Abstract

In this report, we describe the structural characterization of three 2,4-disubstituted-dihydropyrimidine-5-carbonitrile derivatives, namely 2-{[(4-nitrophenyl)methyl]sulfanyl}-6-oxo-4-propyl-1,6-dihydropyrimidine-5-carbonitrile **1**, 4-(2-methylpropyl)-2-{[(4-nitrophenyl)methyl]sulfanyl}-6-oxo-1,6-dihydropyrimidine-5-carbonitrile **2**, and 2-[(2-ethoxyethyl)sulfanyl]-6-oxo-4-phenyl-1,6-dihydropyrimidine-5-carbonitrile monohydrate **3**. An X-ray diffraction analysis revealed that these compounds were crystallized in the centrosymmetric space groups and adopt an L-shaped conformation. One of the compounds (**3**) crystallized with a water molecule. A cyclic motif (R_2_^2^(8)) mediated by N–H···O hydrogen bond was formed in compounds **1** and **2**, whereas the corresponding motif was not favorable, due to the water molecule, in compound **3**. The crystal packing of these compounds was analyzed based on energy frameworks performed at the B3LYP/6-31G(d,p) level of theory. Various inter-contacts were characterized using the Hirshfeld surface and its associated 2D-fingerprint plots. Furthermore, a molecular docking simulation was carried out to assess the inhibitory potential of the title compounds against the human dihydrofolate reductase (DHFR) enzyme.

## 1. Introduction

Pyrimidine moiety was early discovered as an important scaffold in several chemotherapeutic agents [1]. The chemotherapeutic efficacy of pyrimidine-based drugs is attributed to their inhibitory effect on the biosynthesis of vital enzymes responsible for nucleic acids, such as thymidylate synthetase (TSase), thymidine phosphorylase (TPase), dihydrofolate reductase (DHFR), and reverse transcriptase (RTase). Several pyrimidine-based drugs are currently marketed as antineoplastic agents for the treatment of different human cancers [2,3,4,5,6]. Potent antiviral activities against human immunodeficiency viruses (HIV) [7,8,9,10,11,12], herpes simplex virus (HSV) [13], hepatitis B virus (HBV) [14], and SARS-CoV virus [15] have been reported for numerous substituted pyrimidine derivatives. In addition, pyrimidine-based dihydrofolate reductase (DHFR) inhibitors are currently used as clinically useful chemotherapeutic agents [16,17]. Trimethoprim, the prototype antibacterial DHFR inhibitor was discovered to be a potent drug, mainly in the treatment of urinary tract infections [18]. The next generation of DHFR inhibitors was further developed as potent antibacterial drugs for the treatment of resistant respiratory tract infections [19,20,21]. Pyrimidine-based DHFR inhibitors are also employed as efficient antiprotozoal agents for the treatment of malaria [22,23], leishmaniasis [24], and trypanosomiasis [25]. In addition, several pyrimidine-5-carbonitrile derivatives were reported to display marked antimicrobial activities [26,27,28,29,30,31,32].

In the present investigation, we report an in-depth experimental and theoretical study of the structures of three 2,4-disubstituted dihydropyrimidine-5-carbonitrile derivatives in a trial to explore the mechanism of their antimicrobial activity.

## 2. Results and Discussion

### 2.1. Chemical Synthesis

**Scheme 1:**
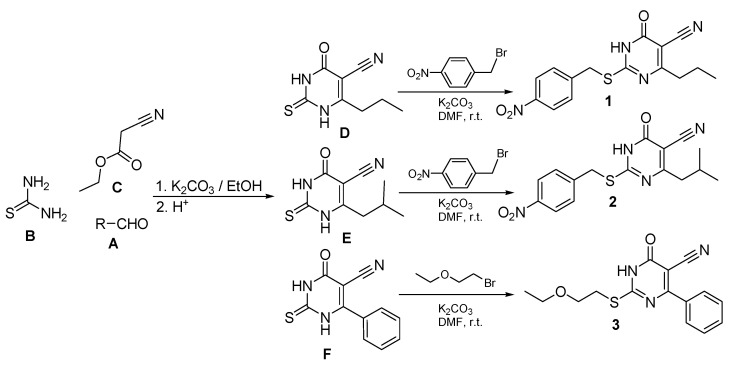
Synthesis of compounds **1**–**3**.

The dihydropyrimidine-5-carbonitriles **1**, **2**, and **3** were synthesized via condensation of the corresponding aldehydes **A** with thiourea **B** and ethyl cyanoacetate **C**, in ethanol, in the presence of anhydrous potassium to yield the intermediate 6-substituted-2-thiouracil-5-carbonitriles **D** [31], **E** [32], and **F** [33]. Compounds **D** and **E** were reacted with 4-nitrobenzyl bromide and compound **F** with 1-bromo-2-ethoxyethane in the presence of anhydrous potassium carbonate to yield the target compounds **1**, **2**, and **3** (Scheme 1).

### 2.2. Crystal Structures

**Figure 1:**
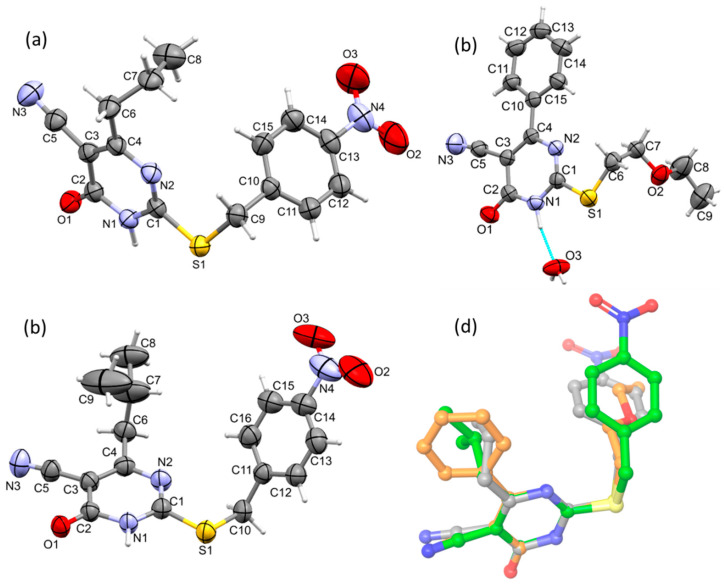
Thermal ellipsoid representation of compounds (**a**) **1**, (**b**) **2**, and (**c**) **3** at 50% probability level with atom numbering scheme is shown, and (**d**) structural superimposition of compounds **1** (grey), **2** (green), and **3** (orange). The water oxygen in compound **3** is not shown for clarity.

Single crystal X-ray diffraction was used to determine the crystal structures of compounds **1***–***3**. A summary of the crystallographic data and structure refinement parameters is listed in Table 1. The Oak Ridge Thermal Ellipsoid Plot (ORTEP) representation at 50% probability corresponding to the asymmetric units of **1**, **2**, and **3** is depicted in Figure 1. The bond lengths and bond angles of the three structures were comparable to the related reported structures [34,35,36,37].

Full crystallographic data for compounds 1 (CCDC 2063317), 2 (CCDC 2063318) and 3 (CCDC 2063320) can be obtained free of charge from The Cambridge Crystallo-graphic Data Centre at: www.ccdc.cam.ac.uk (Supplementary Materials).

Compound **1** crystallized in the monoclinic crystal system with the *P*2_1_/*n* space group. Figure 1a depicts the ORTEP diagram of compound **1**. The asymmetric unit of compound **1** contains one molecule in an L-shape molecular conformation, where the angle between the 4-nitrobenzyl moiety and 1,6-dihydropyrimidine-5-carbonitrile (C9-S1-C1) was found to be 102.37°. The dihedral angle formed between the nitrobenzyl and pyrimidine ring is 87.90°.

**Figure 2:**
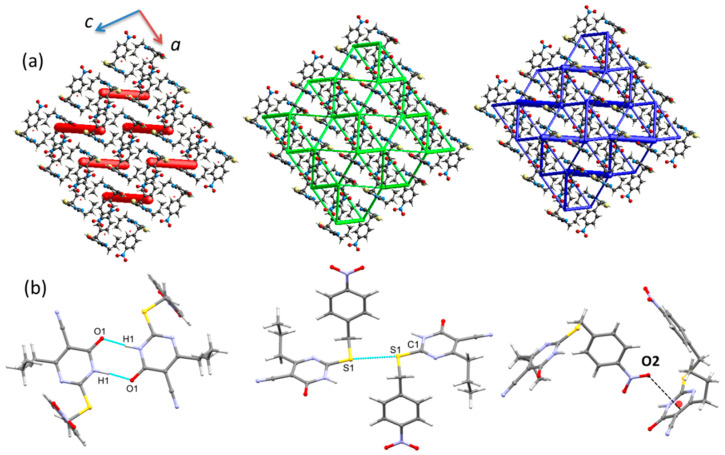
(**a**) Packing diagram of compound **1** viewed along the crystallographic *b*-axis with the incorporation of energy framework (electrostatic energy: red; dispersion energy: green; and net interaction energy: blue with the cylindrical size of 80; interaction energy for molecular pairs less than 15 kJ mol^−1^ has been omitted for clarity) and (**b**) molecular dimers formed in the crystal structure of **1**.

To understand the crystal packing, we created an energy framework that combines pairwise intermolecular interaction energies with a graphical depiction of their magnitude [38]. The energy frameworks of **1** were projected onto the crystallographic *ac* plane, as shown in Figure 2a. As shown from this figure, the molecules of **1** are packed in a columnar fashion. The molecular packing was mainly stabilized by a pair of N1–H···O1 (H···O = 1.795 Å, N···O = 2.800 (2) Å, ∠NHO = 174°) hydrogen bonds which form between the NH and carbonyl groups of two centrosymmetrically-related pyrimidine rings (Figure 2b). These hydrogen bonds led to a cyclic inversion dimer with a R_2_^2^(8) graph-set motif. This hydrogen bond (represented as large cylindrical tubes) links the molecules in the layer with molecules in an adjacent layer. The total intermolecular interaction energy for this hydrogen bonded dimer is −78.2 kJ mol^−1^. In addition to this, a chalcogen bond of the type C1–S1···S1 is present, with the distance and angle 3.363 (2) Å and 166.2 (1)°, respectively (Figure 2b). The role of the chalcogen bond in this structure is similar to that of a N–H···O hydrogen bond. One of the oxygens (O2 atom) of the nitro group makes a short N-O···π (centroid of the pyrimidine ring) interaction with the distance of 3.033 (2) Å (Figure 2b). This interaction also bridges the adjacent ladders. The ladder-pattern is formed by weak van der Waals-type interactions.

Compound **2** crystallized in the monoclinic crystal system with the space group *C*2/*c*. The asymmetric unit contains one molecule, as shown in Figure 1b. The 4-nitrobenzyl moiety and 1,6-dihydropyrimidine-5-carbonitrile are positioned in an L-shaped structure similarly to compound **1**. Molecules **2** are arranged as layers in the solid state, and these layers run parallel to the crystallographic *a* axis. Furthermore, the adjacent layers are interlinked by a short and directional C–H···O interaction (involving H15 from nitrobenzyl and O3 of the nitro group), with H···O = 2.35 Å and ∠CHO = 161° forming a double layer. Moreover, the adjacent layers are also connected by a C– S···S chalcogen bond (S1···S1 = 3.444 (2) Å and ∠C1-S1···S1 = 162 (1)°).

**Figure 3:**
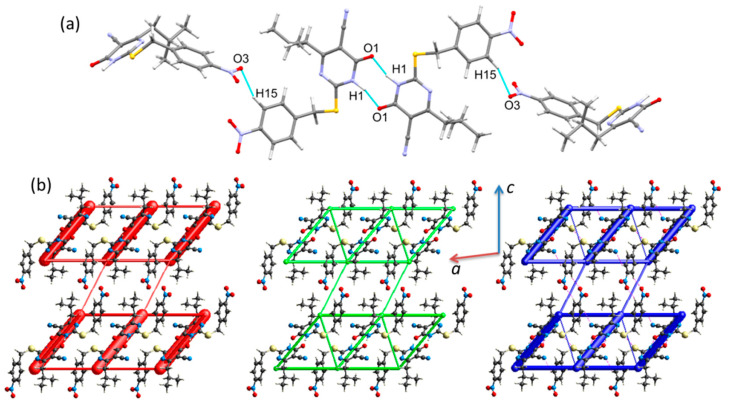
(**a**) Supramolecular chain built by intermolecular N/C–H···O hydrogen bonds, and (**b**) packing diagram of compound **2** viewed along the crystallographic *b*-axis with the incorporation of the energy framework (electrostatic energy: red; dispersion energy: green and net interaction energy: blue with the cylindrical size of 80; interaction energy for molecular pairs less than 10 kJ mol^−1^ has been omitted for clarity).

The adjacent double layers are further interconnected via cyclic N–H···O synthon (involving NH and C=O of pyrimidine ring and R_2_^2^(8) graph-set motif) as observed in **1**. The N–H···O hydrogen bonding geometry (H···O = 1.79 Å, N···O = 2.776 (1) Å and ∠NHO = 163°) is very similar to the structure of **1**. We also noted that the above mentioned C–H···O and N–H···O bonded motifs are connected alternately, leading to the formation of a chain (Figure 3a).

**Figure 4:**
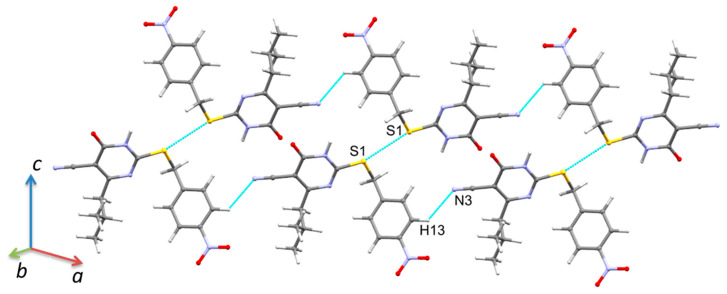
Supramolecular sheet built by intermolecular C–S···S chalcogen bond and C–H···N interaction in compound **2**.

Though the molecular arrangement of **2** in solid-state is somewhat different from the crystal structure of **1**, these two structures show a different 3D-topology of the energy frameworks for electrostatic and net interaction energies (Figure 3b). However, the dispersion energy framework shows a similarity between compounds **1** and **2**. In the electrostatic energy framework, the large vertical cylindrical tubes correspond to cyclic N–H···O hydrogen bonds, and small horizontal tubes bridge the large cylindrical tubes. These horizontal tubes represent the intermolecular C–H···N interaction in which the nitrile N atom acts as an acceptor (C13–H13···N3; H13···N3 = 2.60 Å and ∠CHN = 122°). Furthermore, the adjacent ladder-like topology is interconnected by small vertical cylindrical tubes that represent an intermolecular short and directional C15–H15···O3 interaction. The chalcogen bond observed in this structure interlinks the large vertical cylindrical tubes diagonally in each ladder-like topology and is driven by the dispersion origin. The chalcogen bond and intermolecular C–H···N interaction combined to generate a supramolecular sheet, as shown in Figure 4.

Compound **3** crystallized as a monohydrated form in the triclininc crystal system with the space group *P*-1 (Figure 1c). The asymmetric unit contains one molecule of **3** and one water molecule. Unlike compounds **1** and **2**, compound **3** has a 2-ethoxyethyl group instead of a 4-nitrobenzyl moiety connected to the dihydropyrimidine ring via a thioether bridge. However, compound **3** maintains an L-shape with a bond angle of 101.56° (C8-S1-C12) between the 2-ethoxyethylthio group and the 1,6-dihydropyrimidine-5-carbonitrile. Furthermore, the 4-phenyl group is in the same plane of the 1,6-dihydropyrimidine ring, with a torsional angle of 178.18° (C6-C1-C7-C10). In the asymmetric unit of **3**, the pyrimidine ring NH is involved in an intermolecular N–H···O (N1…O3 = 2.710 Å) hydrogen bond with the water oxygen atom. Due to the presence of the crystallization water molecules in the crystal of **3**, a cyclic N–H···O bonded synthon, as observed in **1** and **2**, disappears.

**Figure 5:**
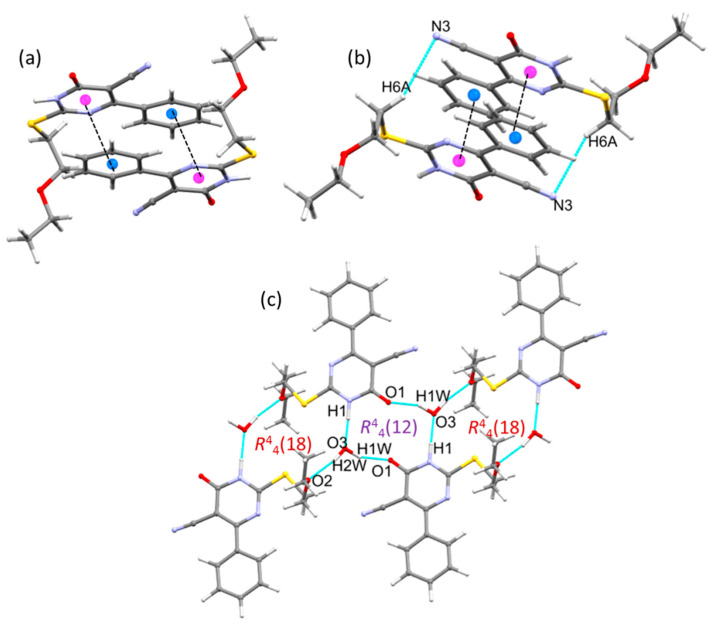
Different dimeric motifs formed by (**a**) π-stacking interaction, (**b**) π-stacking and C–H···N interactions, and (**c**) N–H···O and O–H···O hydrogen bonds in the crystal structure of compound **3**.

The basic packing motif of **3** is the molecular stacking, which forms as a columnar fashion along the *ac* plane, and the water molecules sandwiched between adjacent columns. The molecular dimers formed in this structure are illustrated in Figure 5. The inversion-related molecules (1 − x, 1 − y, 1 − z) of **3** generate molecular stacking, and the centroid-to-centroid distance between the phenyl and pyrimidine rings is 3.588 (2) Å (Figure 5a). Different inversion-related molecules (2 − x, 1 − y, 1 − z) of **3** also produce a molecular stacking, and the centroid-to-centroid separation of phenyl and pyrimidine rings is 3.638 (2) Å. This molecular stacking is further stabilized by the intermolecular C–H···N interaction, in which nitrile N atom acts as an acceptor (Figure 5b).

The water molecule is involved in three intermolecular interactions, of which two are O–H···O type hydrogen bonds and the remaining one is the N–H···O type hydrogen bond (H1···O3 = 1.72 Å, N1···O3 = 2.709 (2) Å and ∠NHO = 167°; symmetry operation: −x + 1, −y + 1, −z + 2). In the O–H···O hydrogen bonds (H1w···O1 = 1.83 Å, O3···O1 = 2.766 (2) Å and ∠OHO = 157°; symmetry operation: −x + 1, −y + 1, −z + 2 and H2w···O2 = 1.86 Å, O3···O2 = 2.832 (2) Å and ∠OHO = 169°; symmetry operation: −x + 1, −y + 2, −z + 2) oxygens of carbonyl and ether moieties are involved as acceptors and water acts as a donor. As shown in Figure 5c, the neighboring molecules of **3** are interlinked by two water molecules, forming alternate *R*^4^_4_(12) and *R*^4^_4_(18) rings.

**Figure 6:**
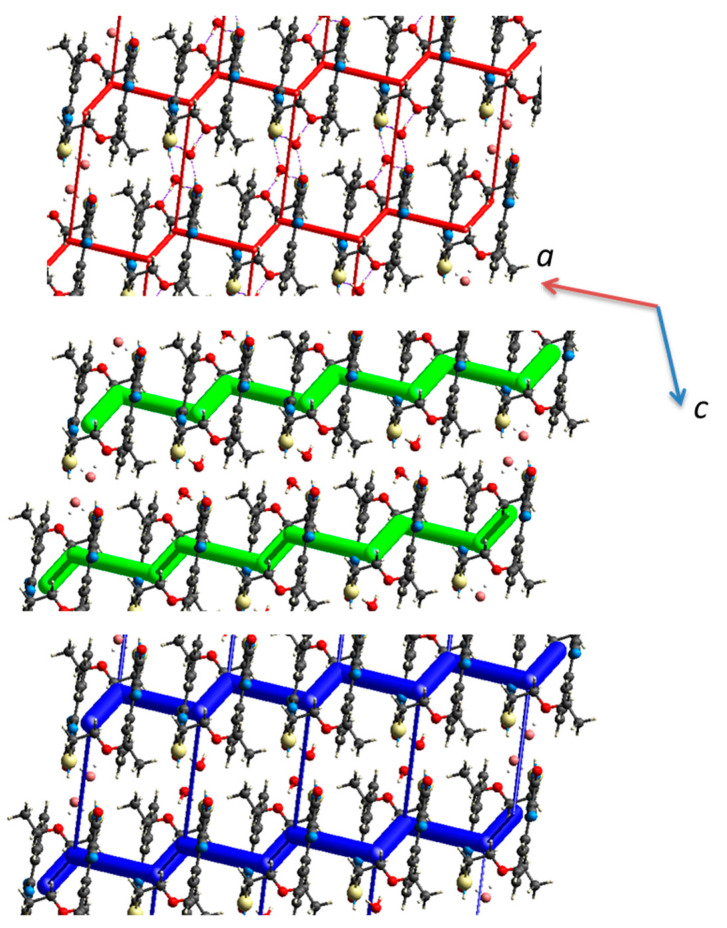
Packing diagram of compound **3** viewed along the crystallographic *b*-axis with the incorporation of the energy framework (electrostatic energy: red; dispersion energy: green; and net interaction energy: blue with the cylindrical size of 80; interaction energy for molecular pairs less than 10 kJ mol^−1^ has been omitted for clarity).

The energy framework of the crystal structure **3** viewed down the *b* axis is depicted in Figure 6. As can be seen from this figure, the crystal packing of compound **3** is predominantly dispersive in nature. The zigzag chains with small cylindrical tubes (electrostatic energy component) run parallel to the crystallographic *a* axis. The adjacent zigzag chains are further interconnected by small vertical tubes. These vertical tubes correspond to intermolecular N–H···O and O–H···O hydrogen bonds. The horizontal tubes represent the π-stacking interaction and C–H···N interaction. Overall, the energy framework of the crystal packing analysis suggests that compounds **1** and **2** display a similar 3D topology of the energy framework, whereas compound **3** shows a different framework compared to the other two compounds. These features suggest that compound **3** may possess different mechanical properties compared to the other two compounds.

### 2.3. Hirshfeld Surface and 2D Fingerprint Plot Analysis

**Figure 7:**
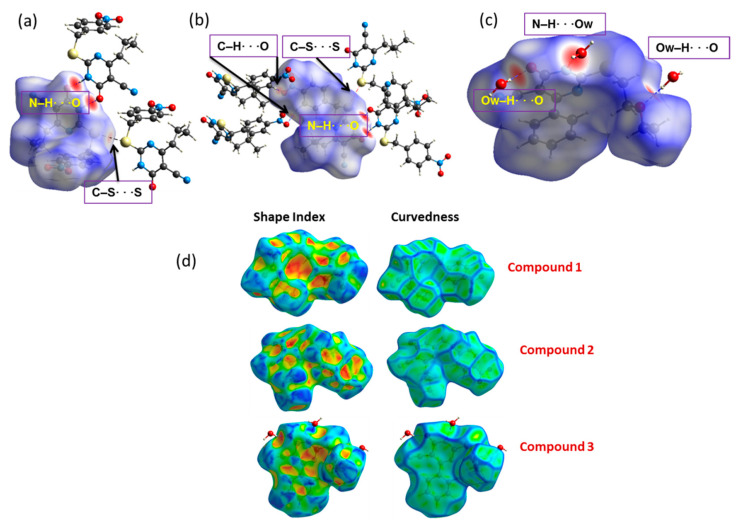
Hirshfeld surface of compounds **1** (**a**), **2** (**b**), and **3** (**c**) mapped with *d*_norm_, and (**d**) shape index, and curvedness.

The Hirshfeld surfaces (HS) of the three dihydropyrimidine derivatives are represented in Figure 7. In compound **1**, the most prominent features are the deep red spots on the HS due to the cyclic N-H···O hydrogen bonds, while a C-S···S type chalcogen bond shows a relatively less intense red spot. In compound **2**, in addition to the above two interactions in **1**, there is a C–H···O interaction in which the nitro oxygen is involved as an acceptor, showing red spots on the HS. Due to the presence of the crystallization water molecule in the crystal of **3**, a cyclic N-H···O synthon has disappeared. However, the water molecule makes three hydrogen bonds, including two Ow–H···O hydrogen bonds with the carbonyl oxygen of pyrimidine and ester oxygen atoms, and the N–H···Ow hydrogen bond with the amine group of the pyrimidine ring. These three intermolecular interactions are shown as intense red areas on the HS.

The shape index of compounds **1** and **2** showed no sign of complementary blue and red triangles on the HS, indicating the absence of π···π interactions in these two compounds. On the other hand, compound **3**, having a phenyl ring at position 4, showed complementary blue and red triangles (at the phenyl ring and dihydropyrimidine rings) on the shape index mapped over the Hirshfeld surface. Furthermore, the curvedness of compound **3** showed a flat region in the same area, confirming the presence of a weak π···π stacking.

**Figure 8:**
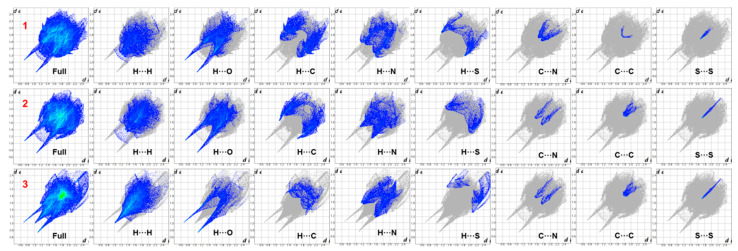
The 2D fingerprint plots for different inter-contacts obtained from structures **1**, **2**, and **3**.

The main intermolecular interactions influencing the molecular packing of compounds **1–3** were studied using the 2D fingerprint plots. The 2D fingerprint plots of the compounds are shown in Figure 8.

It is noticeable that the O···H, N···H, C···H, and S···H contacts play a significant role in crystal packing [39,40,41,42]. The O···H/H···O contacts are represented by a pair of sharp spikes in all three compounds, and these contacts are symmetrical in **1–2** with a *d*_i_ + *d*_e_ distance of ~1.8 Å. In contrast, the corresponding contacts are not symmetrical in the 2D-FP due to the presence of a water molecule in compound **3**. The distribution of H···H contacts markedly varied between compounds **1–2** and **3**. In the latter compound, a single spike with the shortest contact is located at 2.0 Å. We also noted that there is a considerable difference in the contact distribution pattern of C···H/H···C interactions in these compounds. The shortest contact of these interactions is located at ~2.8, 2.7, and 3.0 Å in compounds **1**, **2**, and **3**, respectively, suggesting its strength. The strength of this interaction is nearly equal in **1** and **2**, whereas it is weak in nature in **3**.

Similarly, the distribution pattern of N···H/H···N interactions also looks different in these compounds, with the closest contact separation of ~2.7, 2.6, and 2.8 Å in compounds **1**, **2**, and **3**, respectively. It is important to point out that the contribution (0.7–0.9% to the total HS area) of the chalcogen contact (S···S) is nearly the same in all three compounds. However, there is a remarkable variation in the closest contact distance. The shortest S···S contact is at 3.4 Å in **1** and **2**, and the corresponding contact is located beyond 3.4 Å in **3**, which indicates that the chalcogen bond plays a significant role in the stabilization of the crystal structures of **1** and **2**, rather than the structure of **3**.

**Figure 9:**
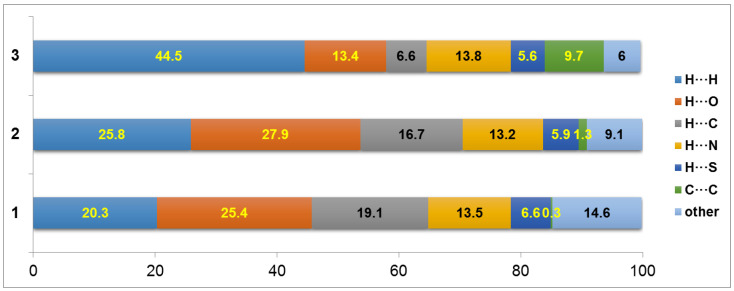
Relative contribution of various intermolecular interactions in the crystal packing of compounds **1, 2**, and **3**.

The relative contribution of various intermolecular interactions in the title dihydropyrimidine derivatives was also obtained from 2D fingerprint plots. In compound **1**, the most important contribution towards crystal packing is from the O···H (25.4%), C···H (19.1%), N···H (13.5%), and S···H (6.6%) contacts. The O···H/H···O showed a dominant role in the overall crystal packing of compound **1** (Figure 9). Compound **2** also showed similar contributions for some of the inter-contacts as compound **1**. The replacement of propyl group with an isobutyl group at position 4 of the dihydropyrimidine ring led to increasing the H···H (25.8%) and O···H (27.9%) and decreasing the H···C (16.7%) contacts compared to compound **1**. Additionally, the nitro group in compound **2** also contributed towards the increase in O···H/H···O contacts in the whole system (Figure 7). Compound 3 has a maximum contribution from H···H (44.5%), followed by N···H (13.8%), O···H (13.4%), and S···H contacts (5.6%). On the other hand, the substitution of the phenyl ring at position 4 of the dihydropyrimidine ring led to a significant increase in the C···C (9.7%) contacts, confirming the π…π interaction in this structure **(Figure 9**).

### 2.4. Molecular Docking Studies as Human Dihydrofolate Reductase (hDHFR) Inhibitors

**Figure 10:**
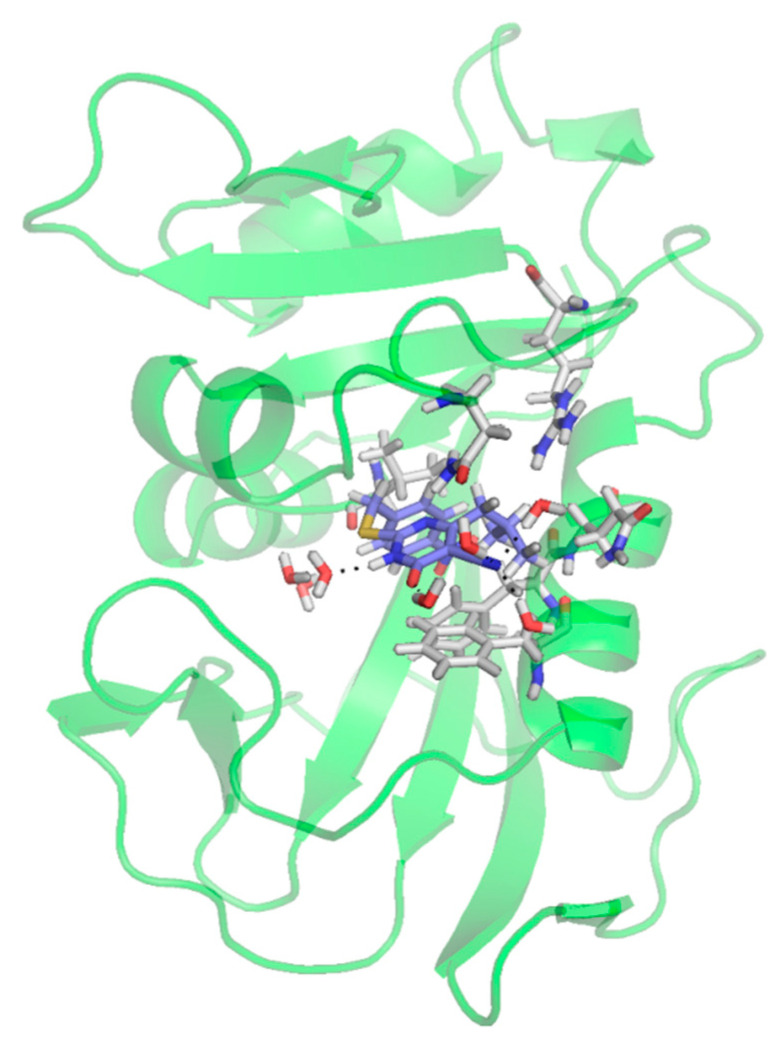
Binding pose of compound 2 (blue) in the active site of hDHFR, with a docking score of -8.58 kcal/mol (hDHFR represented as an illustration in a green color; interacting residues and ligands are represented in a tube model).

**Figure 11:**
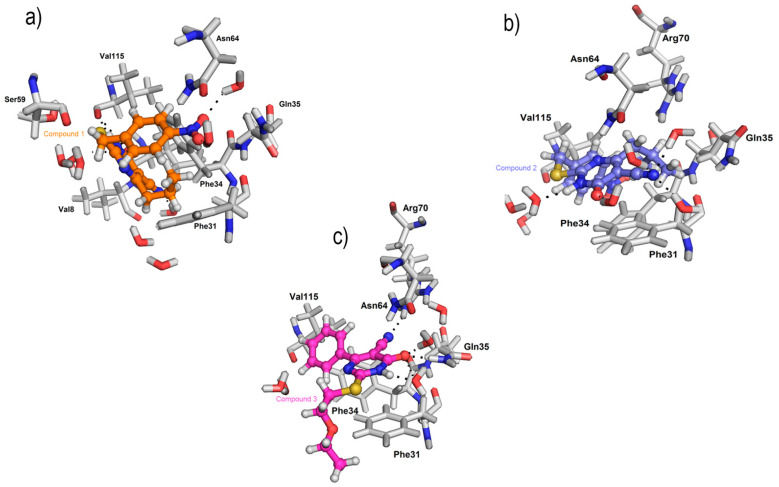
Binding poses of (**a**) compounds **1** (orange), (**b**) **2** (blue), and (**c**) **3** (magenta) in the active site of hDHFR (active site residues and water molecules are represented as tubes and ligands in a ball and stick model).

Molecular docking studies were performed to predict the molecular interaction between the receptor and compounds **1**, **2**, and **3**, and possible binding poses and binding energies. The crystal structures of compounds **1**, **2**, and **3** prepared from LigPrep [43] were subjected to rigid docking against hDHFR using Glide docking, followed by an induced fit docking protocol. Amino acid residues Ile-7, Leu-22, Phe-31, Phe-34, Arg-70, and Val-115 are essential for the activity of hDHFR and DHFR inhibition studies [44]. Our docking results (Table 2) identified compound **2** as a top binding ligand with a docking score of -8.58 kcal/mol (Figure 10). The nitro group formed a π…cation interaction with key residue Phe34, present in the alpha helix. Importantly, the nitrile group and 1,6-dihydropyrimidne enhance the binding energy with several water-mediated hydrogen bonding interactions with Asn64 and Ser59. Compound **1** showed a docking score of -8.34 kcal/mol, it formed key interactions with essential residues Phe34, Val115, and Phe34 in the active site. The 1,6-dihydropyrimidine ring formed a hydrogen bonding interaction with Val115 and π…π interaction with Phe34 residues. Unlike compound **2**, the nitro group of compound **1** formed a π…cation interaction with the Phe31 residue. Additionally, the nitro and nitrile groups enhanced the binding affinity with hDHFR via several water-mediated interactions with Val8, Gln35, and Asp64. Compound **3** showed a docking score of −7.34 kcal/mol, with hydrogen bonding interactions with Arg70 and Gln35 residues in the active site. The oxygen of the carbonyl group formed a hydrogen bond with residue Gln35 and the nitrile group formed an interaction with Arg70. Furthermore, compound **3** and hDHFR complex were stabilized by water-mediated hydrogen-bonding interactions with Asn64 (Figure 11).

## 3. Materials and Methods

### 3.1. Synthesis and Crystallization

Compounds **1**, **2**, and **3** were prepared following the reaction sequences in Scheme 1. The pure single crystals were obtained by slow evaporation of EtOH/CHCl_3_ (1:2, v/v) solution at room temperature to yield the compounds as colorless transparent prism crystals.

2-{[(4-Nitrophenyl)methyl]sulfanyl}-6-oxo-4-propyl-1,6-dihydropyrimidine-5-carbonitrile **1** [31]: Yield: 92%; M.p. 210–212 °C (EtOH); Mol. Formula (Mol. wt.): C_15_H_14_N_4_O_3_S (330.36).

4-(2-Methylpropyl)-2-{[(4-nitrophenyl)methyl]sulfanyl}-6-oxo-1,6-dihydropyrimidine-5-carbonitrile **2** [32]: Yield: 90%; M.p. 217–219 °C (EtOH/H_2_O); Mol. Formula (Mol. wt.): C_16_H_16_N_4_O_3_S (344.39).

2-[(2-Ethoxyethyl)sulfanyl]-6-oxo-4-phenyl-1,6-dihydropyrimidine-5-carbonitrile monohydrate **3** [30]: Yield: 42%; M.p. 161–163 °C (EtOH); Mol. Formula (Mol. wt.): C_15_H_15_N_3_O_2_S (301.36).

### 3.2. Single Crystal X-ray Diffraction Determination

Single crystals of compounds **1**–**3** were used to measure the X-ray diffraction at room temperature (293 K) on a Xcalibur, Ruby, Gemini diffractometer (Agilent Technologies, Inc., Santa Clara, CA, USA) using a single wavelength X-ray source (Cu *K*α radiation: λ = 1.54184 Å). Pre-experiment, data collection, data reduction, and analytical absorption correction were performed with the program suite *CrysAlisPro* (Rigaku Oxford Diffraction) [45]. The structures were solved with the Olex2 program [46], and the refinement was performed with the *SHELXL 2018/3* program [47]. In all three structures, the position of the NH atoms was located from a difference Fourier map, and in compound **3**, the positions of water H atoms were also located from a difference map. In compound **2**, EADP constraints were applied to atoms C7, C8, C14, and C15 and DFIX restraints were applied to C7–C8/C7–C9 bonds with 2.0 Å. Furthermore, SIMU restraints were applied to make the atomic displacement parameter (ADP) values for these atoms more reasonable. In compound **3**, the O–H distances were restrainted to 0.90 (2) Å during the final refinement. In all three structures, H atoms bound to C atoms were placed in geometrically idealized positions (C–H = 0.93–0.98 Å) and were constrained to ride on their parent atoms with *U*_iso_(H) = 1.2*U*_eq_(C). The positions of methyl H atoms were placed in calculated positions (C–H = 0.96 Å), but were allowed to rotate about the C–C bonds and constrained to ride on their parent atoms with *U*_iso_(H) = 1.5*U*_eq_(C).

### 3.3. Computational Details

#### 3.3.1. Hirshfeld Surface Analysis

Hirshfeld surface and 2D fingerprint plots were generated using CrystalExplorer3.1 [42]. The Crystallographic Information Files (cif files) of compounds **1**, **2**, and **3** were used as input files to visualize HS and 2D fingerprint plots. The normalized contact distance (*d*_norm_), mapped throughout the surface, is defined as:

$$d_{norm}=\frac{d_{i}-r_{i}^{vdW}}{r_{i}^{vdW}}+\frac{d_{e}-r_{e}^{vdW}}{r_{e}^{vdW}}$$

where *d_e_* and *d_i_* represent the distances from a point on the surface to the nearest nucleus outside and inside the surface, respectively, and *r^vdW^* corresponds to the van der Waals (vdW) radii of the atoms involved. The red-white-blue colors on the *d_norm_* surface indicate the interatomic distances are shorter than vdW (red), equal to vdW (white), and longer than vdW (blue) [39]. Shape index and curvedness were generated to study π···π interaction in the current derivatives. Complimentary blue and red triangle on the shape index and green flat areas on the curvedness are distinctive features of π···π stacking [40]. Furthermore, 2D-fingerprint plots were used to compare the relative contribution of various non-covalent contacts present in the molecular packing of the dihydropyrimidine derivatives [40,41]. Furthermore, the energy frameworks for compounds **1**–**3** were generated with a B3LYP/6-31G(d,p) level of approximation using the CrystalExplorer-17.5 program [48].

#### 3.3.2. Molecular Docking Studies

##### Protein Preparation and Grid Generation

The 3D structure coordinates of human dihydrofolate reductase (hDHFR) were retried from RCSB (PDB ID: 1drf) [49], and refined using a protein preparation module in Schrödinger suite [50]. In protein preparation, hydrogen atoms were added, water beyond 5 Å was removed, added missing side chain using prime, assigned pH 7.0 ± 1, followed by structure optimization and minimization steps. After the protein preparation, the resulting structure was subjected to a grid generation protocol. Receptor grid was generated on the folic acid binding site using Schrodinger suite to dock small molecules of interest.

##### Ligand Preparation

Structures of compounds **1**–**3**, along with cocrystalized ligand (folic acid), were imported into the Maestro suite for docking calculation. These ligands were subjected to ligand preparation using LigPrep module [51]. Possible ring conformations, ionization states, and tautomers were generated using the Maestro suite.

## 4. Conclusions

The crystal structures of three 2,4-disubstituted-dihydropyrimidine-5-carbonitrile derivatives were determined at the room temperature. The structural analysis revealed that compounds **1** and **2** are primarily stabilized by a N–H···O hydrogen bond and C–S···S chalcogen bond. In compound **2**, a short intermolecular C–H···O interaction and C–H···N interaction provided additional stabilization. The N–H···O hydrogen bond forms an *R*^2^_2_(8) cyclic synthon in both compounds **1** and **2**. Due to the presence of a water molecule in compound **3**, the cyclic synthon disappears, and the water molecule participates in N–H···O and O–H···O hydrogen bonds. In addition, C–H···O and C–H···N interactions are additionally stabilized in compound **3**. The Hirshfeld analysis revealed variations in different inter-contacts due to the presence of substituents in these compounds. The shape index plot confirmed the π-stacking interaction in compound **3**. The energy framework analysis showed that these compounds adopt distinct 3D-energy topologies. However, the dispersion energy framework showed a similar feature in compounds **1** and **2**. Molecular docking analysis indicated that these three compounds showed an inhibitory potential against the human DHFR enzyme.

## Figures and Tables

**Table 1:** Crystal data and structure refinement parameters of compounds **1–3**.

	Compound 1	Compound 2	Compound 3
Empirical formula	C_15_H_14_N_4_O_3_S	C_16_H_16_N_4_O_3_S	C_15_H_17_N_3_O_3_S
Formula weight	330.36	344.39	319.38
Temperature (K)	293 (2)		
Crystal system	Monoclinic	Monoclinic	Triclinic
Space group	*P*2_1_/*n*	*C*2/*c*	*P*-1
a/Å b/Å c/Å	12.5792 (7) 9.5493 (5) 13.3158 (8)	18.3676 (7) 5.7996 (2) 31.8592 (12)	7.1456 (4) 10.8642 (6) 11.2566 (7)
α/° β/° γ/°	90 106.432 (7) 90	90 95.343 (4) 90	104.672 (5) 108.159 (5) 95.421 (5)
Volume/Å3	1534.20 (16)	3379.0 (2)	788.84 (8)
*Z*	4	8	2
Calculated density (g/cm^3^)	1.430	1.354	1.345
Absorption coefficient (mm^−1^)	2.068	1.899	1.968
*F*(000)	688	1440	336
Crystal size (mm^3^)	0.16 × 0.12 × 0.08	0.16 × 0.05 × 0.05	0.26 × 0.14 × 0.10
Radiation	Cu *K*α (λ = 1.54184)		
2Θ range for data collection	8.5 to 151.4	5.6 to 151.4	8.6 to 151.1
Index ranges	−15 ≤ h ≤ 15, −11 ≤ k ≤ 11, −16 ≤ l ≤ 16	−22 ≤ h ≤ 22, −7 ≤ k ≤ 6, −39 ≤ l ≤ 40	−8 ≤ h ≤ 8, −13 ≤ k ≤ 13, −13 ≤ l ≤ 11
Reflections collected	15422	16836	12241
Independent reflections	3163 [R_int_ = 0.0971, R_sigma_ = 0.0654]	3418 [R_int_ = 0.1098, R_sigma_ = 0.0941]	3220 [R_int_ = 0.0442, R_sigma_ = 0.0377]
Data/restraints/parameter	3163/0/213	3418/2/211	3220/2/212
Goodness-of-fit on *F*^2^	1.030	0.998	1.035
Final *R* indices [*I>2* σ*(I)*]	*R*_1_ = 0.0569, w*R*_2_ = 0.1209	*R*_1_ = 0.0766, w*R*_2_ = 0.1777	*R*_1_ = 0.0432, w*R*_2_ = 0.1040
Final *R* indices (all data)	*R*_1_ = 0.1087, w*R*_2_ = 0.1479	*R*_1_ = 0.1565, w*R*_2_ = 0.2358	*R*_1_ = 0.0689, wR_2_ = 0.1210
Largest diff. peak and hole (e.Å^−3^)	0.20/−0.25	0.52/−0.36	0.16/−0.18
CCDC number	2063317	2063318	2063320

**Table 2:** The docking scores of compounds **1**, **2**, and **3** against hDHFR after IFD.

Compound	Docking Score	Glide Energy	No. of Interactions	No. of H-Bonding Residues	Interacting Residues
**1**	−8.53 kcal/mol	−93.57	1	0	Phe34 (pi.cation) Interaction with Gln35, Asn64, and Ser59 via water
**2**	−8.34 kcal/mol	−84.82	1	1	H-bond: Val115, Phe34 (pi…pi), Phe31 (pi…cation) Interaction with Gln35, Asn64, and Val8 via water
**3**	−7.34 kcal/mol	−75.03	2	2	H-bond: Arg 70, and Gln35; water-mediated interactions with Asn4

## Data Availability

Not applicable.

## Supplementary Materials

The Checkcif report of compounds **1**–**3** are available online.

## Sample Availability

Samples of compounds **1**, **2** and **3** are available from the corresponding author.
