# Supplementary material for: Structural Insights of Three 2,4-Disubstituted Dihydropyrimidine-5-carbonitriles as Potential Dihydrofolate Reductase Inhibitors
Source: Molecules. 2021 May 29;26(11):3286. doi: 10.3390/molecules26113286 (PMC8198998; doi:10.3390/molecules26113286)
Supplement: Supplementary file 1 [file molecules-26-03286-s001.zip › molecules-1231826-supplementary.pdf]

Supporting Information

Structural Insights of Three 2,4-Disubstituted Dihydropyrimidine-5-carbonitriles as Potential Dihydrofolate Reductase Inhibitors

Lamya H. Al-Wahaibi [1], Althaf Shaik [2], Mohammed A. Elmorsy [3], Mohammed S. M. Abdelbaky [4], Santiago Garcia-Granda [4], Subbiah Thamotharan [5], Vijay Thiruvenkatam [6] and Ali A. El-Emam [7,*]

Checkcif report for compound 1

Datablock

Bond precision:	C-C = 0.0052 A		Wavelength=1.54184
Cell:	a=12.5792(7)	b=9.5493(5)	c=13.3158(8)
	alpha=90	beta=106.432(7)	gamma=90
Temperature:	293 K		

	Calculated	Reported
Volume	1534.20(16)	1534.20(16)
Space group	P 21/n	P 21/n
Hall group	-P 2yn	-P 2yn
Moiety formula	C15 H14 N4 O3 S	C15 H14 N4 O3 S1
Sum formula	C15 H14 N4 O3 S	C15 H14 N4 O3 S
Mr	330.36	330.36
Dx,g cm-3	1.430	1.430
Z	4	4
Mu (mm-1)	2.068	2.068
F000	688.0	688.0
F000'	691.37	
h,k,lmax	15,12,16	15,11,16
Nref	3194	3194
Tmin,Tmax	0.742,0.848	0.900,1.000
Tmin'	0.718	

Correction method= # Reported T Limits: Tmin=0.900 Tmax=1.000 AbsCorr = MULTI-SCAN

Data completeness= 1.000   Theta(max)= 75.706

R(reflections)= 0.0569(1849)   wR2(reflections)= 0.1479(3163)

S = 1.030   Npar= 213

The following ALERTS were generated. Each ALERT has the format
test-name_ALERT_alert-type_alert-level.
Click on the hyperlinks for more details of the test.

Alert level C

PLAT242_ALERT_2_C Low 'MainMol' Ueq as Compared to Neighbors of N4 Check
PLAT340_ALERT_3_C Low Bond Precision on C-C Bonds .... 0.00515 Ang.

PLAT906_ALERT_3_C Large K Value in the Analysis of Variance ...... 7.900 Check

---

Alert level G

PLAT199_ALERT_1_G Reported _cell_measurement_temperature ..... (K) 293 Check
PLAT200_ALERT_1_G Reported _diffrn_ambient_temperature ..... (K) 293 Check
PLAT883_ALERT_1_G No Info/Value for _atom_sites_solution_primary . Please Do !
PLAT912_ALERT_4_G Missing # of FCF Reflections Above STh/L= 0.600 30 Note
PLAT941_ALERT_3_G Average HKL Measurement Multiplicity .... 4.9 Low
PLAT978_ALERT_2_G Number C-C Bonds with Positive Residual Density. 0 Info

---

0 **ALERT level A** = Most likely a serious problem - resolve or explain
0 **ALERT level B** = A potentially serious problem, consider carefully
3 **ALERT level C** = Check. Ensure it is not caused by an omission or oversight
6 **ALERT level G** = General information/check it is not something unexpected

3 ALERT type 1 CIF construction/syntax error, inconsistent or missing data
2 ALERT type 2 Indicator that the structure model may be wrong or deficient
3 ALERT type 3 Indicator that the structure quality may be low
1 ALERT type 4 Improvement, methodology, query or suggestion
0 ALERT type 5 Informative message, check

Checkcif report for compound 2

Datablock

Bond precision: C-C = 0.0080 A Wavelength=1.54184

Cell:	a=18.3676(7)	b=5.7996(2)	c=31.8592(12)
	alpha=90	beta=95.343(4)	gamma=90
Temperature:	293 K		

	Calculated	Reported
Volume	3379.1(2)	3379.0(2)
Space group	C 2/c	C 2/c
Hall group	-C 2yc	-C 2yc
Moiety formula	C16 H16 N4 O3 S	C16 H16 N4 O3 S1
Sum formula	C16 H16 N4 O3 S	C16 H16 N4 O3 S
Mr	344.39	344.39
Dx,g cm-3	1.354	1.354
Z	8	8
Mu (mm-1)	1.899	1.899
F000	1440.0	1440.0
F000'	1446.88	
h,k,lmax	22,7,40	22,7,40
Nref	3516	3516
Tmin,Tmax	0.898,0.916	0.871,1.000
Tmin'	0.732	

Correction method= # Reported T Limits: Tmin=0.871 Tmax=1.000 AbsCorr = MULTI-SCAN

Data completeness= 1.000 Theta(max)= 75.711

R(reflections)= 0.0766(1660) wR2(reflections)= 0.2358(3418)

S = 0.998 Npar= 211

The following ALERTS were generated. Each ALERT has the format
test-name_ALERT_alert-type_alert-level.
Click on the hyperlinks for more details of the test.

Alert level C

PLAT026_ALERT_3_C Ratio Observed / Unique Reflections (too) Low .. 47% Check
PLAT220_ALERT_2_C NonSolvent Resd 1 C Ueq(max)/Ueq(min) Range 3.9 Ratio
PLAT234_ALERT_4_C Large Hirshfeld Difference C6 --C7 . 0.16 Ang.
PLAT242_ALERT_2_C Low 'MainMol' Ueq as Compared to Neighbors of N4 Check
PLAT242_ALERT_2_C Low 'MainMol' Ueq as Compared to Neighbors of C6 Check
PLAT340_ALERT_3_C Low Bond Precision on C-C Bonds .... 0.008 Ang.
PLAT906_ALERT_3_C Large K Value in the Analysis of Variance ...... 8.039 Check
PLAT906_ALERT_3_C Large K Value in the Analysis of Variance ...... 2.153 Check
PLAT911_ALERT_3_C Missing FCF Refl Between Thmin & STh/L= 0.600 7 Report

Alert level G

PLAT002_ALERT_2_G Number of Distance or Angle Restraints on AtSite 3 Note
PLAT171_ALERT_4_G The CIF-Embedded .res File Contains EADP Records 2 Report
PLAT172_ALERT_4_G The CIF-Embedded .res File Contains DFIX Records 2 Report
PLAT176_ALERT_4_G The CIF-Embedded .res File Contains SADI Records 2 Report
PLAT199_ALERT_1_G Reported _cell_measurement_temperature ..... (K) 293 Check
PLAT200_ALERT_1_G Reported _diffrn_ambient_temperature ..... (K) 293 Check
PLAT860_ALERT_3_G Number of Least-Squares Restraints .... 2 Note
PLAT883_ALERT_1_G No Info/Value for _atom_sites_solution_primary . Please Do !
PLAT912_ALERT_4_G Missing # of FCF Reflections Above STh/L= 0.600 91 Note
PLAT933_ALERT_2_G Number of OMIT Records in Embedded .res File ... 8 Note
PLAT941_ALERT_3_G Average HKL Measurement Multiplicity .... 4.9 Low
PLAT978_ALERT_2_G Number C-C Bonds with Positive Residual Density. 1 Info

0 **ALERT level A** = Most likely a serious problem - resolve or explain
0 **ALERT level B** = A potentially serious problem, consider carefully
9 **ALERT level C** = Check. Ensure it is not caused by an omission or oversight
12 **ALERT level G** = General information/check it is not something unexpected

3 ALERT type 1 CIF construction/syntax error, inconsistent or missing data
6 ALERT type 2 Indicator that the structure model may be wrong or deficient
7 ALERT type 3 Indicator that the structure quality may be low
5 ALERT type 4 Improvement, methodology, query or suggestion
0 ALERT type 5 Informative message, check

Checkcif report for compound 3

Datablock

Bond precision:	C-C = 0.0036 A		Wavelength=1.54184
Cell:	a=7.1456(4)	b=10.8642(6)	c=11.2566(7)
	alpha=104.672(5)	beta=108.159(5)	gamma=95.421(5)
Temperature:	293 K		

	Calculated	Reported
Volume	788.84(9)	788.84(8)
Space group	P -1	P -1

Hall group	-P 1	-P 1
Moiety formula	C15 H15 N3 O2 S, H2 O	C15 H15 N3 O2 S, H2 O
Sum formula	C15 H17 N3 O3 S	C15 H17 N3 O3 S
Mr	319.38	319.38
Dx,g cm-3	1.345	1.345
Z	2	2
Mu (mm-1)	1.968	1.968
F000	336.0	336.0
F000'	337.63	
h,k,lmax	8,13,14	8,13,13
Nref	3264	3265
Tmin,Tmax	0.754,0.821	0.966,1.000
Tmin'	0.571	

Correction method= # Reported T Limits: Tmin=0.966 Tmax=1.000 AbsCorr = MULTI-SCAN

Data completeness= 1.000 Theta(max)= 75.547

R(reflections)= 0.0432(2299) wR2(reflections)= 0.1210(3220)

S = 1.035 Npar= 212

The following ALERTS were generated. Each ALERT has the format
test-name_ALERT_alert-type_alert-level.
Click on the hyperlinks for more details of the test.

Alert level C

PLAT241_ALERT_2_C High 'MainMol' Ueq as Compared to Neighbors of C8 Check
PLAT906_ALERT_3_C Large K Value in the Analysis of Variance ...... 2.891 Check

---

Alert level G

PLAT002_ALERT_2_G Number of Distance or Angle Restraints on AtSite 3 Note
PLAT154_ALERT_1_G The s.u.'s on the Cell Angles are Equal ..(Note) 0.005 Degree
PLAT172_ALERT_4_G The CIF-Embedded .res File Contains DFIX Records 2 Report
PLAT199_ALERT_1_G Reported _cell_measurement_temperature ..... (K) 293 Check
PLAT200_ALERT_1_G Reported _diffrn_ambient_temperature ..... (K) 293 Check
PLAT860_ALERT_3_G Number of Least-Squares Restraints .... 2 Note
PLAT883_ALERT_1_G No Info/Value for _atom_sites_solution_primary . Please Do !
PLAT912_ALERT_4_G Missing # of FCF Reflections Above STh/L= 0.600 37 Note
PLAT933_ALERT_2_G Number of OMIT Records in Embedded .res File ... 1 Note
PLAT941_ALERT_3_G Average HKL Measurement Multiplicity .... 3.8 Low
PLAT978_ALERT_2_G Number C-C Bonds with Positive Residual Density. 2 Info

---

0 **ALERT level A** = Most likely a serious problem - resolve or explain
0 **ALERT level B** = A potentially serious problem, consider carefully
2 **ALERT level C** = Check. Ensure it is not caused by an omission or oversight
11 **ALERT level G** = General information/check it is not something unexpected

4 ALERT type 1 CIF construction/syntax error, inconsistent or missing data
4 ALERT type 2 Indicator that the structure model may be wrong or deficient
3 ALERT type 3 Indicator that the structure quality may be low
2 ALERT type 4 Improvement, methodology, query or suggestion
0 ALERT type 5 Informative message, check
